# GC-MS, LC-MS/MS, Docking and Molecular Dynamics Approaches to Identify Potential SARS-CoV-2 3-Chymotrypsin-Like Protease Inhibitors from *Zingiber officinale* Roscoe

**DOI:** 10.3390/molecules26175230

**Published:** 2021-08-28

**Authors:** Muhammad Sulaiman Zubair, Saipul Maulana, Agustinus Widodo, Ramadanil Pitopang, Muhammad Arba, Maywan Hariono

**Affiliations:** 1 Department of Pharmacy, Faculty of Science, Tadulako University, Palu 94118, Indonesia; saifulmaulana011@gmail.com (S.M.); widodoagustinus@untad.ac.id (A.W.); 2 Department of Biology, Faculty of Science, Tadulako University, Palu 94118, Indonesia; pitopang_64@yahoo.com; 3 Department of Pharmaceutical Chemistry, Faculty of Pharmacy, Halu Oleo University, Kendari 93231, Indonesia; muh.arba@uho.ac.id; 4 Department of Pharmaceutical Chemistry, Faculty of Pharmacy, Sanata Darma University, Yogyakarta 55282, Indonesia

**Keywords:** *Zingiber officinale*, LC-MS/MS, Steroids, 24-Methylcholesta-7-en-3β-on, 3CL Protease, SARS-CoV-2

## Abstract

This study aims to identify and isolate the secondary metabolites of *Zingiber officinale* using GC-MS, preparative TLC, and LC-MS/MS methods, to evaluate the inhibitory potency on SARS-CoV-2 3 chymotrypsin-like protease enzyme, as well as to study the molecular interaction and stability by using docking and molecular dynamics simulations. GC-MS analysis suggested for the isolation of terpenoids compounds as major compounds on methanol extract of pseudostems and rhizomes. Isolation and LC-MS/MS analysis identified 5-hydro-7, 8, 2′-trimethoxyflavanone (**9**), (*E*)-hexadecyl-ferulate (**1**), isocyperol (**2**), *N-*isobutyl-(2*E*,4*E*)-octadecadienamide (**3**), and nootkatone (**4**) from the rhizome extract, as well as from the leaves extract with the absence of **9**. Three known steroid compounds, i.e., spinasterone (**7**), spinasterol (**8**), and 24-methylcholesta-7-en-3*β*-on (**6**), were further identified from the pseudostem extract. Molecular docking showed that steroids compounds **7**, **8**, and **6** have lower predictive binding energies (MMGBSA) than other metabolites with binding energy of −87.91, −78.11, and −68.80 kcal/mole, respectively. Further characterization on the single isolated compound by NMR showed that **6** was identified and possessed 75% inhibitory activity on SARS-CoV-2 3CL protease enzyme that was slightly different with the positive control GC376 (77%). MD simulations showed the complex stability with compound **6** during 100 ns simulation time.

## 1. Introduction

COVID-19, caused by the SARS-CoV-2 virus, is a global pandemic that has negatively impacted human life in this recent time. As of July 2021, approximately 196 million people have been infected, and 4.2 million have died from this disease [1]. The absence of medicine has encouraged the application of several synthetic drugs to be repurposed for combating the virus replication, such as hydroxy chloroquine and remdesivir. However, the attention for adverse side effect prompts us to find drugs that are effective and selective in inhibiting the replication of the virus [2,3]. To date, several targets of SARS-CoV-2 virus have been identified, such as 3 chymotrypsin-like protease, papain-like protease, RNA dependent RNA polymerase, and spike-glycoprotein, which have afforded significant research on discovering and developing new drugs focusing on the viral spreading inhibition. 3 Chymotrypsin-like protease (3CLpro) SARS-CoV-2 has become an interesting target, as if it is inhibited, the viral replication process will be disrupted by inhibiting the viral polyproteins cleavage [4].

Natural sources have been becoming one of options to discover such drugs since their biological diversity provides more interesting chemical skeletons and diverse structures [5,6]. The mixture of plants from Zingiberaceae family, known as medicinal herbs of “empon-empon”, has been suddenly consumed by javanese in Indonesia to fight against COVID-19 during this pandemic time as they believe that this traditional herb can cure the pneumonia-like symptoms in COVID-19 patients. Zingiberaceae plants are also well and broadly distributed in several cities in Indonesia as they are commonly utilized for daily food, spicing, and medication. *Zingiber officinale* Roscoe (ginger) is drawing attention to be studied as this plant showed a broad pharmacological activity, such as anti-bacterial, anti-oxidant, anti-tumor, and anti-inflammatory [7,8,9,10]. Respiratory disorders, diabetes mellitus, obesity, cardiovascular, and neurodegenerative defects were also reported among diseases that can be treated using ginger [11,12,13,14,15]. Mostly, these activities were reported to possess the such effects due to the content of phenolic compounds, such as gingerols, shogaols, and paradols. The aqueous extract was reported to inhibit avian influenza virus H_9_N_2_ activity, and its lyophilized water decoction extract was able to inhibit the proliferation of human respiratory syncytial virus in human respiratory tract cell lines [16,17]. The docking molecular study of its main metabolites on 3 chymotrypsin-like protease SARS-CoV-2 receptor suggested their potency as antivirus for treating COVID-19 [18].

In this present study, the isolation and identification of the bioactive compounds of *Z. officinale* (leaves, pseudostems and rhizomes), have been performed using combination of chromatographic/spectroscopic analysis and in silico methods. Many studies have reported this approach as successful strategy to discover potential bioactive compounds from the nature, as well as to determine their binding interaction mode on protein target [19,20,21]. Gas chromatography-mass spectrophotometry (GC-MS) was used to identify the major compounds for further isolation, liquid chromatography-mass spectrophotometry/mass spectrophotometry (LC-MS/MS) was used to identify the isolated compounds, and nuclear magnetic resonance (NMR) was used to elucidate the chemical structure of isolated compound. Meanwhile, molecular docking was used to predict the potential compounds that can inhibit SARS-CoV-2 3CL protease at the molecular level. To confirm the activity, an in vitro assay of the isolated compound against the SARS-CoV-2 3CL protease enzyme were also carried out to prove the insilico concept. Further molecular dynamics analysis was also performed to study the stability of the isolated compound as complex with SARS-CoV-2 3CLprotease. To the best of our knowledge, this study is the first report on SARS-CoV-2 3CL protease inhibitory activity of compound from this plant.

## 2. Results

### 2.1. Extraction and GC-MS Analysis of Z. officinale Methanol Extract

**Figure 1:**
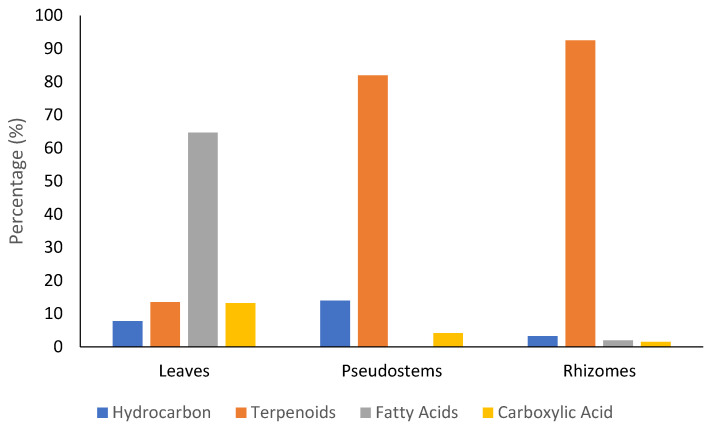
Diversity of secondary metabolites on *Z. officinale* methanol extract identified by GC-MS.

The maceration extraction method has resulted the methanol extract of leaves, pseudostems, and rhizomes with the yields 1.44%, 8.87%, and 19.81%, respectively. The methanol extracts were then analyzed by GC-MS (Shimadzu, Kyoto, Japan) for the identification of chemical compounds (Supplementary Materials). It can be seen from Figure 1 that the terpenoids was found as the most abundant compounds on the pseudostems and rhizome parts with the percentage of 81.96% and 92.51%, respectively. Meanwhile, in leaves, it was dominated by fatty acids with the percentage of 64.66%, and terpenoid was found with only 13.47%. This result suggested for the isolation of terpenoid compounds from each part of plants. Liquid-liquid extraction was then applied by using solvents with different polarity (*n*-hexane, ethyl acetate, and water), and *n*-hexane extract was chosen for the next isolation process based on detected terpenoids compounds that are mostly a class of terpenes and sesquiterpenes.

### 2.2. Isolation and LC-MS/MS Identification of Isolates from Z. officinale n-Hexane Extract

The *n*-hexane extracts of *Z. officinale* leaves, pseudostems and rhizomes were fractionated on a vacuum-liquid chromatography (VLC) column, with silica gel 60 and eluted by a different polarity of solvents from *n*-hexane-dichloromethane-ethyl acetate to methanol. The VLC fractions having the same profiles were combined each other to collect 21, 17, and 18 fractions from leaves, pseudostems, and rhizomes extract, respectively. Based on the identification of terpenoids compounds (assigned by the purplish color after spraying with Liebermann-Burchard reagent), the fraction number 14 (leaves), number 7 (pseudostems), and number 46 (rhizomes) were further isolated using preparative-TLC on silica gel GF_254_ to possess a single isolated compound from each fraction and then characterized them using LC-MS/MS (Supplementary Materials).

The LC-MS/MS successfully screened and identified several compounds that were selected based on the similarity percentage of their retardation time (R_t_) and molecular mass with the database from UNIFI 1.8 software. The isolated compounds from leaves and rhizomes showed the same compounds that are (*E*)-hexadecyl-ferulate (**1**), isocyperol (**2**), *N*-Isobutyl-(2*E*,4*E*)-octadecadienamide (**3**), and nootkatone (**4**) with the R_t_ of 9.96, 9.45, 9.53, and 9.38 minutes, respectively. A flavonoid compound, 5-hydro-7,8,2′-trimethoxyflavanone (**9**), was detected on rhizomes but it was absent in leaves. Meanwhile, the isolated compounds from pseudostems were identified as three known steroids, namely 24-methylcholesta-7-en-3*β*-on (**6**), spinasterone (**7**), and spinasterol (**8**), with the R_t_ of 10.25, 10.29, and 10.37 minutes, respectively (Table 1).

### 2.3. Molecular Docking

The LC-MS/MS identified compounds from *Z. officinale n*-hexane extracts (leaves, pseudostems, and rhizomes) were then subjected for molecular docking simulations to predict the potential compounds that can inhibit the SARS-CoV-2 3 CL protease. The optimized structures were docked to the viral protease binding site, which can be seen in Table 2. It showed that three known steroid compounds identified from the pseudostem part have lower predictive binding energies of molecular mechanics-generalized Born surface area (MMGBSA) than other compounds, including the co-crystallized ligand, and indinavir as the positive controls. Interestingly, **7**, **8**, and **6** exhibited binding energies with the value of −87.41, −78.11, and −68.80 kcal/mol, respectively, much lower than two positive controls, including baicalein (−47.14 kcal/mol) and remdesivir (−68.55 kcal/mol). Although indinavir showed binding energy slightly lower (−76.44 kcal/mol) than **6**, it is higher than **7** and **8**, indicating that compounds **6**–**8** are at least comparable with the positive controls in binding to the 3CLpro. The more negative value of this energy will exhibit the lower free energy along with the stronger binding. Therefore, the isolated steroid compounds were suggested for further purification and NMR analysis to elucidate the molecular structure, as well as to confirm the activity on SARS-CoV-2 3CL protease enzyme.

**Figure 2:**
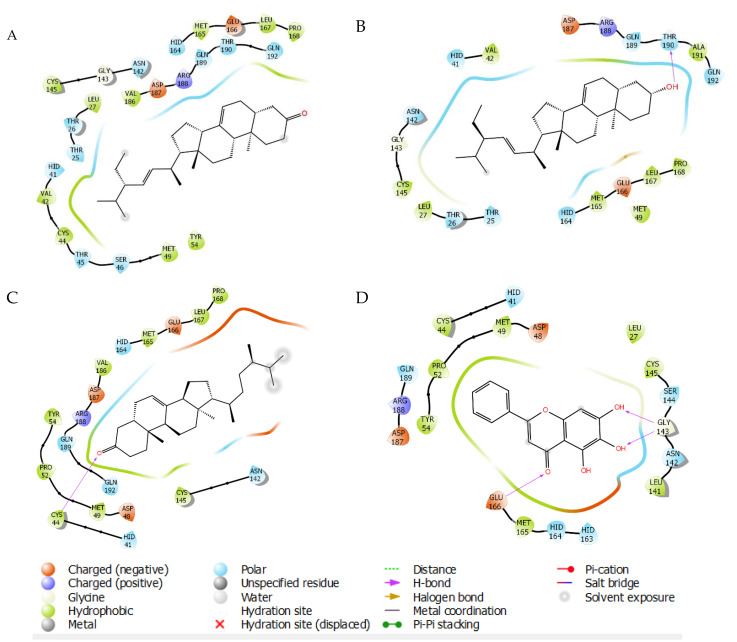
Molecular interactions of SARS-CoV-2 3CL protease of **7** (**A**), **8** (**B**), **6** (**C**) dan baicalein (**D**) as co-crystallized inhibitor.

SARS-CoV-2 3CL protease in complex with baicalein was chosen as the protein model based on the characteristic of this such ligand having more drug-like structure than peptidomimetic compound, which is commonly used as a protease inhibitor. Re-docking analysis of this co-crystallized nonpeptidomimetic inhibitor on the binding site of the viral protease (PDB 6m2n) represents the similar pose with the reported X-ray crystallography, in which hydrogen bonding (H-bond) interaction was found between the carbonyl group of baicalein and Glu166, as well as the multiple H-bond between the two phenolic hydroxyl groups and Gly143. Hydrophobic interactions were found between the free phenyl ring of baicalein with Met49, Cys44, Pro52, and Tyr54 (Figure 2D) [22,23]. The steroid compounds showed binding modes mimicking the baicalein by polarly interacting with Glu166 and Gly143. The unique interaction was found only in the hydroxyl group of **8** through H-bond interaction with Thr190 (Figure 2B). Meanwhile, the only carboxyl group of **6** interacts via H-bond with Cys44 (Figure 2C). Definitely, there is no H-bond interaction found on **7** complexes with the 3CLpro. However, hydrophobic interaction was observed on the residues of Val 42, Cys 44, Leu 167, and Pro 168, giving an extra affinity to bind with the 3CLpro (Figure 2A).

### 2.4. NMR Analysis and SARS-CoV-2 3CL Protease Inhibitory Activity Verification

The steroid compounds isolated from the pseudostem part of *Z. officinale* were purified by successive TLC preparative and underwent NMR analysis that led to structure elucidation of **6**, based on ^1^H and ^13^C-NMR spectral data that can be seen in Table 3.

**Figure 3:**
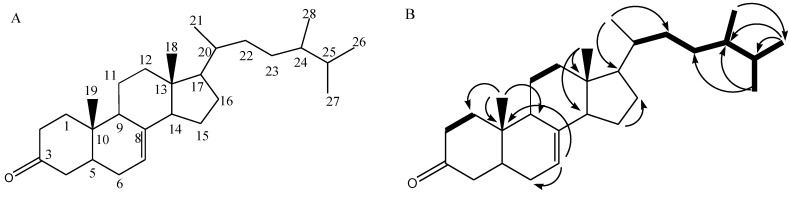
Molecular structure of 24-methylcholesta-7-en-3*β*-on (**A**) and its H-H COSY and HMBC correlation (**B**).

Compound **6** was isolated as a colorless powder. Its molecular mass was based on LC-MS/MS analysis with *m*/*z* at 399.36180 [M + H]^+^ and molecular formula of C_28_H_46_O. The ^13^C NMR data (Table 3) supported the chemical structure with 28 carbon atom signals categorized by Distortionless Enhancement by Polarization Transfer (DEPT) experiment into six methyls, ten methylenes, eight methines, and four quaternary carbons. Six degrees of unsaturation, calculated from the molecular formula, were attributed to a carbonyl group (δ_C_ 204.6 ppm) assigned for C-3, a vinylic system at C-7 and C-8 (δ_C_ 140.1 and 123.7 ppm), and four rings of steroidal skeleton. The ^1^H NMR (Table 3) showed six methyl signals characteristic for cholesterol-type steroids skeleton, at δ_H_: 0.70 and 1.17 ppm, assigned for two methyls at C-18 and C-19, and four doublet signals for a methyl at δ_H_ 0.79, 0.81, 0.90, and 1.00 ppm for C-28, C-21, C-26, and C-27, respectively. A broad singlet at δ_H_ 5.71 was for olefinic proton H-7. From the ^1^H-^1^H COSY correlation, a spin system between H-C1 and H-C2 was observed and connected by long-range C-H correlation (HMBC) between Me-19 (δ_H_ 1.17, br *s*) and C-1 (35.6), C-9 (53.8), and C-10 (38.6) established the closing of ring A. The connection with ring B was supported by long-range C-H correlation (HMBC) between H-C7 (δ_H_ 5.71, *s*) and C-6 (32.9) and C-10 (38.6). The connection to the ring C was proven by the ^1^H-^1^H COSY correlation between H-C11 and H-C12 and supported by the long-range C-H correlation (HMBC) between Me-18 (δ_H_ 0.70 ppm, s) and C-13 (42.2)/C-14 (39.6). The connection between steroid skeleton and its side chain was observed by HMBC long-range correlation between Me-21 (δ_H_ 0.81 ppm, *d*) and C-17 (δ_C_ 48.5)/C-22 (29.1). Furthermore, the consecutive protons were observed from H-C-22, H-C-23, H-C-24, H-C-25, H-C-26, H-C-27, and H-C-28. Long-range C-H correlation (HMBC) was observed between Me-28 and C-26 (18.7), between Me-26 and C-24 (55.9), and C-25 (36.0), between Me-27 and C-23 (40.5) and C-24 (55.9) (Figure 3, Supplementary Materials).

**Figure 4:**
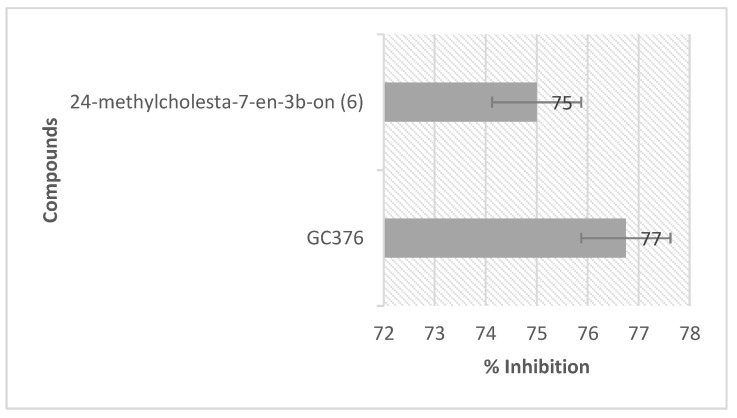
The % inhibition of 24-methylcholesta-7-en-3β-on (**6**) (200 µg/mL~500 µM) against SARS-CoV-2 3CLpro by in vitro assay. GC376 (100 µM) was used as the positive control and the experiment was triplicated.

Compound **6** was tested on SARS-CoV-2 enzyme inhibition and found the percentage of inhibition as 75% at the concentration of 200 µg/mL (Figure 4). This in vitro assay used the foster resonance energy transfer (FRET) principle, worked by measuring the fluorescence of the donor bead in the fluorogenic substrate upon cleaving under proteolysis by the 3CLpro [24]. The fluorescence will be reduced by the presence of an inhibitor, describing the inhibitory activity of such an inhibitor toward the protease. Biological activity verification showed that this compound demonstrated enzymatic inhibition by 75% (500 µM) against the SARS-CoV-2 3CLpro enzyme. Although it is still less potent than the positive control (GC376, 77% at 100 µM), there is still a hope to proceed a series of concentration of compound 6 to calculate its real IC_50_. Unfortunately, due to our resource limitations, we have not performed this experiment yet.

### 2.5. Molecular Dynamics (MD) Simulation

**Figure 5:**
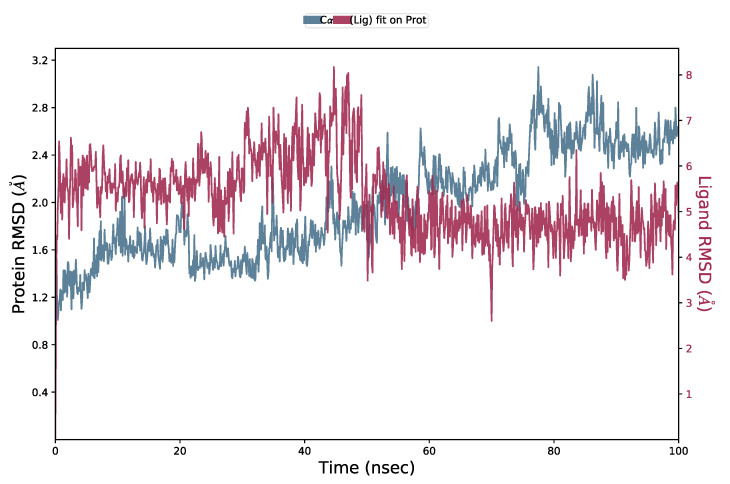
RMSD analysis of MD simulation trajectory (blue = RMSD of the Cα protein; red = RMSD of the ligand-bound protein). The RMSD plot obtained for **6** on SARS-CoV-2 3CL protease complex (PDB ID 6m2n).

MD simulations were performed to further analyze the complex stability of steroid compound **6** with the viral protease active site. The result can be seen in Figure 5. **6** was found to be relatively stable during 100 ns of the simulation times by distinction of RMSD complex ligand-protein and Cα protein below 3 Å.

**Figure 6:**
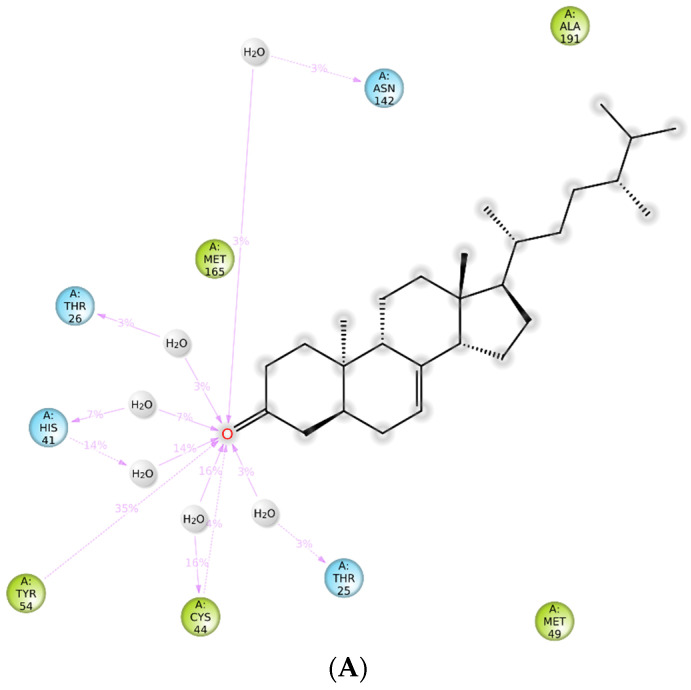

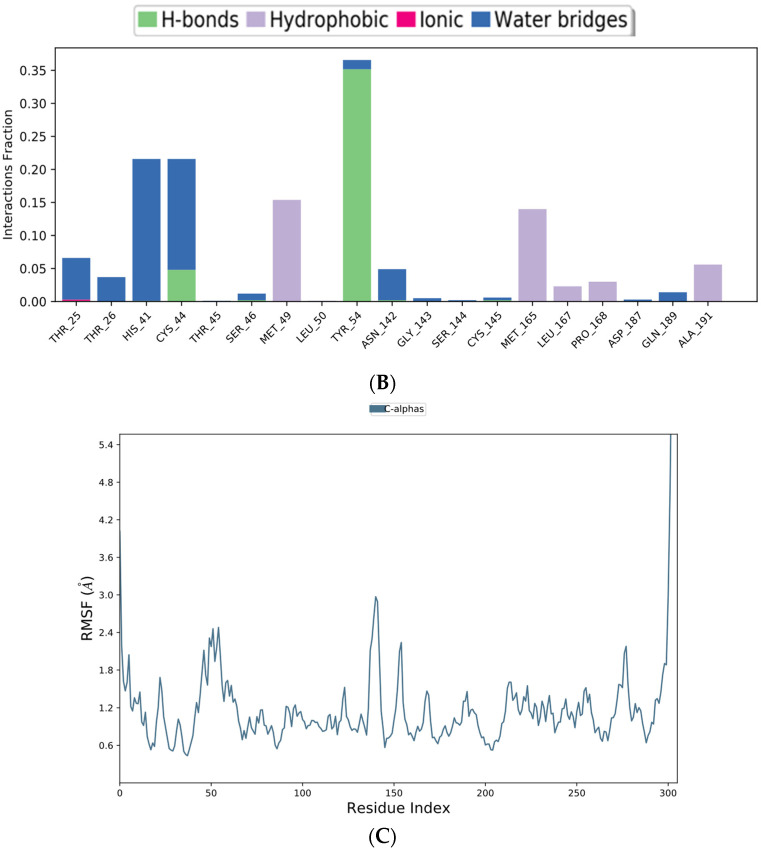
Molecular interaction (**A**), bonding type (**B**), and RMSF plot (**C**) of compound **6** with SARS-CoV-2 3CL protease after 100 ns MD simulation.

Figure 6A shows the residue interactions of viral protease with the steroid compound **6**. It shows that H-bond between carboxyl group of **6** and Cys44 was retained during the simulation time of 100 ns. Meanwhile, hydrophobic interaction keep occurs with Met49, Met165, Leu167, Pro168, and Ala191. Figure 6B shows how **6** interacted mostly through H-bonds with Tyr54 and Cys44. Furthermore, Root Mean Square Fluctuation (RMSF) was used to evaluate the stability of the ligand with the specific amino acid residues of the SARS-CoV-2 3CLpro catalytic site, which are His41 and Cys145 [23]. Figure 6C shows that **6** might bind to catalytic sites both His41 and Cys145 with RMSF 0.755 Å and 0.880 Å, respectively. The lowest RMSF with the specific amino acid residues could reflect the most stable interaction leading to the marker of such amino acids in the 3CLpro catalytic site.

## 3. Discussion

Many bioactive compounds from ginger has been identified so far, mostly constituted by phenolics and terpenoids compounds [25]. The GC-MS analysis of methanol extract of *Z. officinale* identified terpenoids as major compounds in the pseudostems and rhizomes parts. Meanwhile, in leaves, the terpenoids were found as the second after fatty acids. Among the terpenoids, zerumbone was detected with the high percentage of 36.04%, 15.25%, and 2.25% on rhizomes, pseudostems, and leaves, respectively. Terpenoids, including steroids, were reported to possess potential antiviral activities, such as anti-hepatitis B, anti-HIV-1, hepatitis C, and anti-Herpes simplex virus [26,27,28]. Several studies also suggest the potency of terpenoids/steroids as SARS-CoV-2 3CL protease inhibitors [29,30,31,32,33]. Based on both chromatographic and spectroscopic data, the isolated compounds from our study were confirmed having terpenoids/steroids structure, such as **2** and **6**.

The prediction for anti-SARS-CoV-2 on the identified compounds was performed by in silico methods employing docking molecular as a fast and less time-consuming strategy in the drug discovery process. Interestingly, steroid compounds **6**, **7**, and **8** were found to be more potential in inhibiting SARS-CoV-2 3CL protease enzymes than others. To date, there is no report for the presence of the steroid compound **6**, **7**, and **8** on *Z. officinale* extract. Compound **6** was reported as main sterol of *Polyporus sulfureus*. Meanwhile, compound **7** and **8** were reported from *Samanea saman* [34,35]. For biological activity, only compound **8** was reported to possess an antiproliferative effect on HeLa and RAW 264.7 cervical cancer cell lines [36]. The ability of steroids to inhibit the protease SARS-CoV-2 enzyme was also reported by Narkedhe et al. (2020), in which *β*-sitosterol, a common steroid from plant, was proposed by computational docking as inhibitor for main protease SARS-CoV-2. However, in vitro examination on SARS-Coronavirus 3CLpro showed weak inhibition with the IC_50_ of 1210 µM [32,37]. Further purification and characterization of the isolated compound by utilizing NMR analysis was successful in elucidating the chemical structure of **6**. Biological activity verification showed that this compound demonstrated enzymatic inhibition by 75% against the SARS-CoV-2 3CLpro enzyme, slightly less than the positive control (GC376). Molecular dynamics simulation during 100 ns showed that **6** was more stable in complex with the viral protease. Its H-bond interactions mediated by water molecule with the His41 and Cys145 were retained during the 100 ns simulation which are in line with the reference ligand. This enzymatic inhibition supported the insight on molecular mechanism predicted by molecular docking and molecular dynamics simulations results suggesting that this approach can be used for discovering potential drug compounds from nature. The inhibitory potency of other isolates and the extract must be further tested and continued by in vivo and clinical studies to support the application as medicine for COVID-19 disease.

## 4. Materials and Methods

### 4.1. General

TLC aluminium sheets 20 × 20 cm silica gel 60 F254, silica gel 60 (Merck, Darmstadt, Germany), and pre-coated TLC glass plates SIL G-25 UV254, 0.25 mm silica gel (Sigma, St. Louis, MO, USA) were used for thin layer chromatography analysis, vacuum liquid column chromatography, and preparative TLC, respectively. Spots on TLC were visualized by using spraying reagent of Liebermann Burchard for terpenoid/steroid detection. Shimadzu QP-2010 Gas Chromatograph Mass Spectrometer Ultra (Shimadzu, Kyoto, Japan) was used for GC-MS analysis. Acquity UPLC I-Class System with the XEVO G2-XS QTof Mass Spectrometer (Waters, Milford, MA, USA) was used for LC-MS/MS analysis, and NMR JEOL ECZ-500 and Variant Unity INOVA-500 Spectrometer (Agilent Technologies, Santa Carla, CA, USA) were used to elucidate the structural compounds. The nuclear magnetic resonance (NMR) was recorded at 500 MHz for ^1^H and 150 MHz for ^13^C. Chemical shifts are given in δ (ppm) relative to TMS as internal standard, and deuterated chloroform was used as the solvent. The SARS-CoV-2 3CLpro #78042 assay kit was purchased from BPS Bioscience Inc., (San Diego, CA, USA).

### 4.2. Plant Material

The *Z. officinale* plant was collected from Banggai Regency, Central Sulawesi, Indonesia, in December 2019. The plant was identified in Sulawesi Biodiversity Unit-Tadulako University (Herbarium Celebense) with the voucher specimen number 118/UN.28.UPT-SDHS/LK/2019 and deposited at the Herbarium.

### 4.3. Extraction and Isolation

The whole part of the plant (1290 g of leaves, 310 g of pseudostem, and 368 g of rhizomes) was extracted by maceration method using methanol (14 L) for 3–5 days. The collected filtrate was evaporated to obtain viscous methanolic extract (18.58 g for leaves, 27.52 g for pseudostem, and 72.9 g for rhizome). The methanolic extract of each part of the plant (10 g) was further separated by applying liquid-liquid extraction using *n*-hexane:water (1:1) and successively continued by using ethyl acetate:water (1:1) to separately collect the *n*-hexane, soluble, and insoluble ethyl acetate fractions. The *n*-hexane fraction (each 3 g) was poured into a vacuum column chromatography system packed by silica gel (60–120 mesh). Gradient system of solvent, starting from *n*-hexane 100%, *n*-hexane/dichloromethane mixture, dichloromethane/ethyl acetate mixture and ended by ethyl acetate 100%, ethyl acetate/methanol, and then methanol 100%, was used to elute the crude fractions. A total of 78 fractions for leaf, 153 fractions for pseudostem, and 105 fractions for rhizome were collected. TLC analysis was used to identify the similar fractions, affording 21 fractions for leaf, 17 fractions for pseudostem, and 18 fractions for rhizome. The fraction 14 (leaves, 260 mg), 7 (pseudostems, 230 mg), and 46 (rhizomes, 320 mg), which was found to contain terpenoid/steroids, further isolated using preparative TLC with *n*-hexane:ethyl acetate (1:9) as a mobile phase. Isolation of fraction 14 (leaves) generated chromatogram where the third band with R_f_ 0.46 (brown color with sulfuric acid-methanol) was taken to give the first isolate as colorless powder (10.5 mg). Likewise, isolation of fraction 7 (pseudostem) shows R_f_ 0.50 (brown color with sulfuric acid-methanol) was taken to give the second isolate as colorless powder (15.2 mg). Finally, isolation of fraction 46 (rhizome) demonstrates the first band with R_f_ 0.46 (brown color with sulfuric acid-methanol) was taken to give the third isolate as colorless powder (12.4 mg), as well. All isolated compounds were further analyzed by using LC-MS/MS. The isolated compound from fraction 7 (pseudostem) was further purified by successive preparative TLC using n-hexane:chloroform (5:2) as the mobile phase. The single spot at TLC (R_f_ 0.71) was collected to give compound **6** (3.99 mg; yield 1.73%).

### 4.4. GC-MS Analysis

Methanol extract of leaves, pseudostems, and rhizomes of *Z. officinale* were analyzed by GC-MS (Shimadzu QP-2010 Gas Chromatograph Mass Spectrometer Ultra), equipped by an autosampler AOC-20i and capillary column (SH-Rxi-5Sil MS) with the diameter 30 m × 0.25 mm × 0.25 μm. Helium was used as carrier gas (1.0 mL/min), with temperature injection of 250 °C; splitless mode; a column oven temperature of 70 °C at the beginning and held for 2 min, and then ramped to 200 °C at the rate of 10 °C/min and end temperature 280 °C and held for 9 min at the rate 5 °C/min; an MS ion source temperature of 200 °C, and an interface temperature of 280 °C, were set. The secondary metabolites were identified by comparing the experimental molecular mass spectra and base peak of each chromatogram with the Wiley and NIST database libraries.

### 4.5. LC-MS/MS Analysis

Each of the isolated compounds (1 mg) was dissolved in 1 mL of methanol (LC-MS chromasolv^®^ grade). One microliter aliquot of the sample was injected into the column. Formic acid 0.1% (*v*/*v*) in water and acetonitrile plus formic acid 0.1% (*v*/*v*) were used as gradient solvent for eluting the sample that initially started by ratio 95:5, continued by ratio 60:40 from 1.00 to 8.00 min, ratio 0:100 from 8.00 to 13.00 min, and at the end with the ratio 95:5. The flowing rate is 0.3 mL/min. The instrument was set as follows: acquisition time 0.00–16.00 min, start mass 50.00–1200.00 *m*/*z*, scan time 0.100 s, low CE 6 eV, high CE 10–40 eV, cone voltage 30 V, collision energy 6 eV; acquisition mode ESI (+), capillary voltage 2 kV, source temperature 120 °C, desolvation temperature 500 °C, cone gas flow 50 L/h, and desolvation gas flow 1000 L/h, sample temperature 20 °C, and column temperature 40 °C. The data obtained were processed by using UNIFI software (version 1.8, Waters Corporation, Milford, MA, USA) with a screening solution workflow, which helped in automated data processing for reporting the positive identifications. The result was compared with database that collected more than 1200 compounds based on chemical structure, molecular formula, and molecular mass from various web-based resources.

### 4.6. Molecular Docking

For the molecular docking, the protein used was the crystal structures of SARS-CoV-2 3CL protease with the pdb code 6m2n [23]. The protein was optimized by Protein Preparation Wizard module in Maestro Schrödinger 2020-3 software (Schrödinger, New York, NY, USA) [38,39]. The missing hydrogens were added during optimization process, and partial charges were also assigned using OPLS_2005 forcefield. Moreover, hydrogens and heavy atoms in protein were prepared in restrained minimization state. The 2D structures of *Z. officinale* compounds identified from LC-MS/MS analysis were converted to 3D structures using Maestro Schrödinger 2020-3 by LigPrep Module and OPLS_2005 forcefield with pH adjusted 7.4 via Epik [40]. LigPrep facilitated the protonation, tautomeric, and ionization states of each compound, as well as correct proper bond orders. In order to specify the docking region, the grid box was distinguished by selecting co-crystallized ligand of protease receptor to maintain that the center of docked compounds is in a similar dimension with the binding box. Docking protocol was run in extra precision (XP) mode through Glide using OPLS_2005 forcefield with flexible ligand and rigid receptor conditions [41,42]. To evaluate the potential of each ligand as 3CL protease inhibitor of SARS-CoV-2, molecular mechanics-generalized Born surface area (MM-GBSA) was used for scoring the docked pose of the ligand [21,43,44].

### 4.7. SARS-CoV-2 3CL Protease In Vitro Inhibition Assay

The assay buffer was prepared by adding 12 μL 0.5 M DTT for a total of 6 mL of assay buffer, which then would be furtherly used. The enzyme and the substrate were each diluted separately by adding, respectively, 3.95 mL and 950 μL of the previously prepared assay buffer (with DTT). The sample for the assay was prepared by dissolving it in DMSO at 100-fold concentration than the final or required concentration in a 96-well microplate. The final concentration of the sample was 200 µg/mL~500 µM. Inhibitor control used in this assay was a peptidomimetic called GC376 diluted in 200 μL water for 500 μM solution. The assay was carried by adding them in the following order: 30 μL of enzyme (5 ng/μL), required volume of sample or inhibitor (GC376), and assay buffer (with DTT) (if necessary) to a total volume of 40 μL. The initial mixture was incubated for 30 min at 25 °C with slow shaking, and then followed by the addition of 10 μL of substrate (250 μM) for a mixture with the final volume of 50 μL. The mixtures were then incubated overnight and measured with Synergy HTX-3 Multi-mode Reader (Winooski, VT, USA) at 360/460 nm.

### 4.8. Molecular Dynamics Simulation

A molecular dynamics (MD) study was conducted using the Desmond module in Schrödinger software (Version) [45,46,47]. Before commencing the MD process, the system was constructed by selecting ligand-protein complexes and submerged them into an SPC (simple point charge) water box at 10 Å. Moreover, to the system was added Counter ions (33 Na^+^, and 29 Cl^−^ ions), to neutralize charges; additionally, Salts ions (sodium and chloride) were also set to 0.15 M to approximate physiological conditions. The MD simulation was conducted in NPT conditions (temperature 300 K and pressure 1.63 bar) for 50 ns with recording intervals set to 1.2 ps for energy and 20 ps for trajectory. Afterward, the MD simulations were run with the OPLS_2005 forcefield.

## 5. Conclusions

GC-MS, LC-MS/MS analysis, and docking molecular simulations were successfully used to identify the potential compounds from *Zingiber officinale* plant with the activity as inhibitor for SARS-CoV-2 3CL protease enzyme. Compound **6**, **7**, and **8** were steroid class compounds, found in pseudostem part, that showed low values of predictive binding energy (MMGBSA). Further purification and NMR characterization led to the structure of **6** that showed inhibitory activity 75%, slightly less than the positive control GC376 (77%). Further molecular dynamics simulation showed that **6** was found to be more stable during 100 ns molecular dynamics simulation.

## Figures and Tables

**Table 1:** Compounds identified by LC-MS/MS from the isolate of *Z. officinale* n-hexane extract.

*n*-Hexane Extract of	Compound’s Code	Compounds Identified	R_t_ (min)	[M + H]^+^
Leaves	**1**	(*E*)-Hexadecyl-ferulate	9.96	419.3156
	**2**	Isocyperol	9.45	221.1910
	**3**	*N*-Isobutyl-(2*E*,4*E*)-octadecadienamide	9.53	336.3268
	**4**	Nootkatone	9.38	219.1752
	**5**	Candidate mass C_22_H_45_NO	10.04	340.3577
Pseudostems	**6**	24-methylcholesta-7-en-3*β*-on	10.25	399.3618
	**7**	Spinasterone	10.29	411.3616
	**8**	Spinasterol	10.37	413.3770
Rhizomes	**1**	(*E*)-hexadecyl-ferulate	9.96	419.3156
	**2**	Isocyperol	9.45	221.1910
	**3**	*N*-Isobutyl-(2*E*,4*E*)-octadecadienamide	9.53	336.3268
	**4**	Nootkatone	9.38	219.1752
	**9**	5-hydro-7,8,2′-trimethoxyflavanone	9.25	329.1026

**Table 2:** Docking molecular of identified metabolites on SARS-CoV-2 3CL protease with baicalein as the native ligand (pdb code 6m2n).

Identified Compounds	Compound’s Code	MMGBSA Binding Energy (kcal/mol)
Spinasterone	**7**	−87.41
Spinasterol	**8**	−78.11
24-methylcholesta-7-en-3*β*-on	**6**	−68.80
*N*-Isobutyl-(2*E*,4E)-octadecadienamide	**3**	−65.44
5-hydro-7,8,2′-trimethoxyflavanone	**9**	−65.42
(*E*)-hexadecyl-ferulate	**1**	−65.26
Isocyperol	**2**	−62.04
Nootkatone	**4**	−53.24
Baicalein	-	−47.14
Indinavir	-	−76.44
Remdesivir	-	−68.55

**Table 3:** ^1^H (500 MHz) and ^13^C NMR (150 MHz) spectral data of 6 (CDCl_3_).

P.	δC (*m*)	δH (*m*)
1	35.6 *t*	1.65–1.71 (*m*); 1.98–2.03 (*m*)
2	39.5 *t*	1.98–2.03 (*m*)
3	204.6 *d*	-
4	33.9 *t*	2.26–2.31 (*m*, 1H); 2.40–2.42 (*m*, 1H)
5	55.8 *t*	1.00–1.02 (*m*)
6	32.9 *t*	1.27–1.28 (*d*); 2.23–2.24 (*d*)
7	123.7 *d*	5.71 (*s*, 1H)
8	140.9 *s*	-
9	53.8 *d*	0.95 (*m*)
10	38.6 *s*	-
11	20.9 *t*	1.00–1.01 (*d*); 1.52–1.53 (*d*)
12	32.0 *t*	1.81–1.84 (*m*)
13	42.2 *s*	-
14	39.6 *d*	1.12 (*m*)
15	25.4 *t*	1.21 (*m*)
16	29.7 *t*	1.27 (*m*)
17	45.8 *d*	0.88 (*m*)
18	11.9 *q*	0.70 (*s*,3H)
19	17.4 *q*	1.17 (*s*, 3H)
20	31.9 *d*	1.81–1.84 (*m*)
21	19.8 *q*	0.81–0.82 (*d*, 3H)
22	29.1 *d*	1.28 (*m*)
23	40.5 *t*	1.22 (*m*, 2H)
24	55.9 *d*	1.07 (*m*, 1H)
25	36.0 *d*	1.24 (*m*, 1H)
26	18.7 *q*	0.90–0.91 (*d*, 3H)
27	19.5 *q*	1.00–1.01 (*d*, 3H)
28	20.1 *q*	0.79–0.82 (*d*, 3H)

## Data Availability

Data is contained within the article or supplementary material.

## Supplementary Materials

The following are available online. Table S1. GC-MS analysis of leaves, pseudostems and rhizomes of *Z. officinale* methanol extract, Figure S1. The GC-MS spectral data of *Z. officinale* leaves (A). pseudostems (B) and rhizomes (C) of methanol extract, Figure S2: The TLC chromatogram of fraction number 14, 7, and 46 of *Z. officinale* leaves (A). pseudostems (B) and rhizomes (C) of *n*-hexane extract. The spots were detected under UV light 254 nm (left), and after spraying with Liebermann-Burchard reagent (right). The isolated spots were circled out upon the chromatograms, Figure S3: LC-MS/MS spectral data of compounds isolated from leaves of *Z. offiicinale* n-hexane extract. A. (*E*)-Hexadecyl-ferulate, B. Isocyperol, C. N-Isobutyl-(2*E*,4*E*)-octadecadienamide, D. Nootkatone, E. Candidate mass C_22_H_45_NO, Figure S4: LC-MS/MS spectral data of compounds isolated from pseudostem of *Z. offiicinale* n-hexane extract. A. Spinasterone, B. Spinasterol, C. 24-methylcholesta-7-en-3*β*-on, Figure S5: LC-MS/MS spectral data of compounds isolated from rhizome of *Z. offiicinale* n-hexane extract. A. (*E*)-Hexadecyl-ferulate, B. 5-hydro-7,8,2′-trimethoxyflavanone C. Isocyperol, D. N-Isobutyl-(2*E*,4*E*)-octadecadienamide, E. Nootkatone, Figure S6: ^1^H-NMR spectral data of 24-methylcholesta-7-en-3*β*-on, Figure S7: ^13^C-NMR data of 24-methylcholesta-7-en-3*β*-on, Figure S8: DEPT spectral data of 24-methylcholesta-7-en-3*β*-on, Figure S9: HSQC spectral data of 24-methylcholesta-7-en-3*β*-on, Figure S10: H-H COSY spectral data of 24-methylcholesta-7-en-3*β*-on. Figure S11: HMBC spectral data of 24-methylcholesta-7-en-3*β*-on.
