# Supplementary material for: GC-MS, LC-MS/MS, Docking and Molecular Dynamics Approaches to Identify Potential SARS-CoV-2 3-Chymotrypsin-Like Protease Inhibitors from Zingiber officinale Roscoe
Source: Molecules. 2021 Aug 28;26(17):5230. doi: 10.3390/molecules26175230 (PMC8434146; doi:10.3390/molecules26175230)
Supplement: Supplementary file 1 [file molecules-26-05230-s001.zip › molecules-1346289-supplementary.pdf]

GC-MS, LC-MS/MS, Docking and Molecular Dynamics Approaches to Identify Potential SARS-CoV-2 3-Chymotrypsin-like Protease Inhibitors from *Zingiber officinale* Roscoe

Muhammad Sulaiman Zubair[1,*], Saipul Maulana[1], Agustinus Widodo[1], Ramadanil Pitopang[2], Muhammad Arba[3] and Maywan Hariono[4,*]

1 Department of Pharmacy, Faculty of Science, Tadulako University, Palu, Indonesia, 94118 ; sulaiman_zubair80@yahoo.co.id, sulaimanzubair@untad.ac.id (M.S.Z); saifulmaulana011@gmail.com (S.M); widodoagustinus@untad.ac.id (A.W)
2 Department of Biology, Faculty of Science, Tadulako University, Palu, Indonesia, 94118 ; pitopang_64@yahoo.com
3 Department of Pharmaceutical Chemistry, Faculty of Pharmacy, Halu Oleo University, Kendari, Indonesia, 93231 ; muh.arba@uho.ac.id
4 Department of Pharmaceutical Chemistry, Faculty of Pharmacy, Sanata Darma University, Yogyakarta, Indonesia, 55282 ; mhariono@usd.ac.id

* Correspondence: sulaiman_zubair80@yahoo.co.id, sulaimanzubair@untad.ac.id (M.S.Z); Tel.: (+62)85242083654; mhariono@usd.ac.id (M.H); Tel.: (+62)89506286901

Table of contents	Page
Table S1. GC-MS analysis of leaves, pseudostems and rhizomes of *Z. officinale* methanol extract	3
Figure S1. The GC-MS spectral data of *Z. officinale* leaves (A). pseudostems (B) and rhizomes (C) of methanol extract.	8
Figure S2. The TLC chromatogram of fraction number 14, 7, and 46 of *Z. officinale* leaves (A). pseudostems (B) and rhizomes (C) of *n*-hexane extract. The spots were detected under UV light 254 nm (left), and after spraying with Liebermann-Burchard reagent (right). The isolated spots were circled out upon the chromatograms	9
Figure S3. LC-MS/MS spectral data of compounds isolated from leaves of *Z. offiicinale* n-hexane extract. A. (*E*)-Hexadecyl-ferulate, B. Isocyperol, C. N-Isobutyl-(2*E*,4*E*)-octadecadienamide, D. Nootkatone, E. Candidate mass $C_{22}H_{45}NO$	10
Figure S4. LC-MS/MS spectral data of compounds isolated from pseudostem of *Z. offiicinale* n-hexane extract. A. Spinasterone, B. Spinasterol, C. 24-methylcholesta-7-en- 3*β*-on	11
Figure S5. LC-MS/MS spectral data of compounds isolated from rhizome of *Z. offiicinale* n-hexane extract. A. (*E*)-Hexadecyl-ferulate, B. 5-hydro-7,8,2'-trimethoxyflavanone C. Isocyperol, D. N-Isobutyl-(2*E*,4*E*)-octadecadienamide, E. Nootkatone.	12
Figure S6. ^{1}H-NMR spectral data of 24-methylcholesta-7-en-3*β*-on	13
Figure S7. ^{13}C-NMR data of 24-methylcholesta-7-en-3*β*-on	14
Figure S8. DEPT spectral data of 24-methylcholesta-7-en-3*β*-on	15
Figure S9. HSQC spectral data of 24-methylcholesta-7-en-3*β*-on	16
Figure S10. H-H COSY spectral data of 24-methylcholesta-7-en-3*β*-on	17
Figure S11. HMBC spectral data of 24-methylcholesta-7-en-3*β*-on	18

Table S1. GC-MS analysis of leaves, pseudostems and rhizomes of *Z. officinale* methanol extract

No	Identified Compounds	Class	Peak area (%)	Retardation Time (Rt)
	**Leaves**			
1	Undecane	HC	1.37	6.605
2	1,6-methanonaphthalen-1(2h)-ol	Trp	1.05	15.043
3	Docosanoic acid, methyl ester	FA	1.57	15.433
4	2,6,10-cycloundecatrien-1-one	Trp	2.25	15.716
5	3-n-heptyl-7-methyl-9-(2,6,6-trimethylcyclohex-1-enyl)nona-2,4,6,8-tetraenal	Trp	0.78	15.967
6	Methyl 9-methyltetradecanoate	FA	0.78	16.18
7	Tridecanoic acid, 12-methyl-, methyl ester	FA	0.95	16.276
8	4-hexen-1-ol, 6-(2,6,6-trimethyl-1-cyclohexenyl)-4-methyl-, (E)-	Trp	1.98	16.636
9	2-pentadecanon, 6,10,14-trimethyl-	HC	0.99	16.867
10	11-hexadecenoic acid, methyl ester	FA	1.12	17.687
11	Hexadecanoic acid, methyl ester	FA	21.78	17.945
12	Cycloprop[e]indene-1a,2(1h)-dicarboxaldehyde, 3a,4,5,6,6a,6b-hexahydro-5,5,6b-trimethyl-	Trp	1.18	18.133
13	Dibutyl phthalate	CA	0.85	18.458
14	Hexadecanoic acid, ethyl ester	Trp	3.61	18.88
15	Alloaromadendrenoxid-(1)	Trp	1.88	19.006
16	Cyclopropaneoctanoic acid, 2-hexyl-, methyl ester	FA	1.02	19.151
17	9,12-octadecadienoic acid (z,z)-, methyl ester	FA	12.53	20.343
18	9-octadecenoic acid (z)-, methyl ester	FA	13.92	20.433
19	9-octadecenoic acid, methyl ester	FA	5.27	20.508
20	2-hexadecen-1-ol, 3,7,11,15-tetramethyl-, [r-[r*,r*-(e)]]-	HC	5.46	20.599
21	Octadecanoic acid, methyl ester	FA	5.72	20.812
22	Naphthalene, decahydro-1,1,4a-trimethyl-6-methylene-5-(3-methyl-2,4-pentadienyl)-,	Trp	0.74	23.426
23	1,2-benzenedicarboxylic acid, mono(2-ethylhexyl) ester	CA	9.81	26.979

24	2-pentyl 6-(4-pentylphenyl) 2,6-naphthalenedicarboxylate	CA	1.15	32.908
25	2-pentyl 6-(4-pentylphenyl) 2,6-naphthalenedicarboxylate	CA	2.22	33.123
	Total		99.98	
	**Pseudostems**			
1	Undecane	HC	5.17	6.63
2	1,4,8-cycloundecatriene, 2,6,6,9-tetramethyl-, (E,E,E)-	Trp	1.4	12.181
3	Cyclopropanecarboxylic acid, 2,2-dimethyl-3-(2-propenyl)-, ethyl ester, trans	CA	1	12.618
4	2(4h)-benzofuranone, 5,6,7,7a-tetrahydro-4,4,7a-trimethyl-	Trp	1.16	13.229
5	1,7-dioxaspiro[5.5]undec-2-ene	HC	1.03	13.275
6	1,6,10-dodecatrien-3-ol, 3,7,11-trimethyl-	Trp	18.88	13.5
7	Caryophyllene oxide	Trp	4.68	13.756
8	Caryophyllene oxide	Trp	11.17	13.869
9	Naphthalene, decahydro-4a-methyl-1-methylene-7-(1-methylethenyl)-, [4ar-(4a.alpha.,7.alpha.,8a.beta.)]-	Trp	3.07	14.07
10	2,5,9-trimethylcycloundeca-4,8-dienone	Trp	1.6	14.219
11	4-hydroxy-.beta.-ionone	Trp	1.48	14.298
12	Tetracyclo[6.3.2.0(2,5).0(1,8)]tridecan-9-ol, 4,4-dimethyl-	Trp	4.39	14.557
13	Limonene oxide, trans-	Trp	1.53	14.667
14	Isosteviol methyl ester	Trp	3.09	14.778
15	Longifolenaldehyd	Trp	2.73	14.942
16	3-buten-2-one, 4-(4-hydroxy-2,2,6-trimethyl-7-oxabicyclo[4.1.0]hept-1-yl)-	Trp	5.52	15.105
17	2,6,10-cycloundecatrien-1-one, 2,6,9,9-tetramethyl-, (E,E,E)-, Zerumbone	Trp	15.25	15.709
18	2(4h)-benzofuranone, 5,6,7,7a-tetrahydro-6-hydroxy-4,4,7a-trimethyl-, (6s-cis)-, Loliolide	Trp	3.95	16.275
19	4-hexen-1-ol, 6-(2,6,6-trimethyl-1-cyclohexenyl)-4-methyl-, (E)-	Trp	1.2	16.635
20	2,6,10-trimethyl,14-ethylene-14-pentadecne	HC	1.83	16.796
21	2-pentadecanon, 6,10,14-trimethyl-	HC	2.96	16.919
22	4,6,6,7,8,8-hexamethyl-1,3,4,6,7,8-hexahydrocyclopenta[g]isochromene	Trp	0.86	17.025
23	3,7,11,15-tetramethyl-2-hexadecen-1-ol	HC	1.84	17.122

24	2,6,10-trimethyl,14-ethylene-14-pentadecne	HC	1.09	17.367
25	1,2-benzenedicarboxylic acid, mono(2-ethylhexyl) ester	CA	3.13	27.099
	Total		100.01	
	**Rhizomes**			
1	Eucalyptol	Trp	0.2	5.571
2	1,6-octadien-3-ol, 3,7-dimethyl-	HC	0.2	6.646
3	1,6-octadien-3-ol, 3,7-dimethyl-	HC	0.71	6.707
4	Bicyclo[2.2.1]heptan-2-one, 1,7,7-trimethyl-, (1R)-	Trp	0.78	7.5
5	Bicyclo[2.2.1]heptan-2-one, 1,7,7-trimethyl-, (1R)-	Trp	1.6	7.551
6	Bicyclo[2.2.1]heptan-2-ol, 1,7,7-trimethyl-, (1s-endo)-, Borneol	Trp	0.23	7.954
7	3-cyclohexene-1-methanol, .alpha.,.alpha.4-trimethyl-	HC	0.37	8.306
8	Bicyclo[2.2.1]heptan-2-ol, 1,7,7-trimethyl-, acetate, (1s-endo)-, Borneol acetate	Trp	0.24	9.667
9	Cyclohexane, 1-ethenyl-1-methyl-2,4-bis(1-methylethenyl)-, [1s-(1.alpha.,2.beta.,4.beta.)]-	Trp	0.26	11.196
10	Camphene glycol	Trp	0.2	11.293
11	1H-Cycloprop[e]azulene, 1a,2,3,4,4a,5,6,7b-octahydro-1,1,4,7-tetramethyl-, [1aR-(1a.alpha.,4.alpha.,4a.beta.,7b.alpha.)]-	Trp	0.26	11.492
12	Caryophyllene	Trp	3.54	11.688
13	1,4,7,-cycloundecatriene, 1,5,9,9-tetramethyl-, Z,Z,Z-	Trp	14.56	12.25
14	3-furanacetic acid, 4-hexyl-2,5-dihydro-2,5-dioxo-	CA	0.37	12.402
15	1,6-cyclodecadiene, 1-methyl-5-methylene-8-(1-methylethyl)-, [s-(E,E)]-, GERMACRA-1(10),4(15),5-TRIENE	Trp	0.22	12.521
16	Eudesma-4(14),11-diene	Trp	0.16	12.633
17	6.alpha.-cadina-4,9-diene, (-)-	Trp	0.21	12.701
18	1,3,3-trimethyl-2-oxabicyclo[2.2.2]octan-6-ol	Trp	0.17	12.837
19	4-pentyl-1-(4-propylcyclohexyl)-1-cyclohexene	HC	0.64	12.926
20	4-pentyl-1-(4-propylcyclohexyl)-1-cyclohexene	HC	0.78	12.965
21	3,7-cyclodecadien-1-one, 3,7-dimethyl-10-(1-methylethylidene)-, (E,E)-	Trp	0.28	13.139
22	Cyclohexane, 1-methyl-2,4-bis(1-methylethenyl)-, (1.alpha.,2.beta.,4.beta.)-	Trp	1.57	13.264

23	Cyclohexanemethanol, 4-ethenyl-.alpha.,.alpha.,4-trimethyl-3-(1-methylethenyl)-, [1R-(1.alpha.,3.alpha.,4.beta.)]-	Trp	0.22	13.364
24	1,6,10-dodecatrien-3-ol, 3,7,11-trimethyl-	Trp	0.86	13.437
25	1,4-dimethyl-3-(2-methyl-1-propenyl)-4-vinyl-1-cycloheptene	Trp	0.14	13.558
26	2,5,9-trimethyl-4,8-cycloundecadien-1-one	Trp	0.3	13.754
27	(-)-5-oxatricyclo[8.2.0.0(4,6)]dodecane,,12-trimethyl-9-methylene-, [1R-(1R*,4R*,6R*,10S*)]-	Trp	3.39	13.881
28	Tricyclo[2.2.1.0(2,6)]heptane, 1,7-dimethyl-7-(4-methyl-3-pentenyl)-	Trp	0.15	13.958
29	Naphthalene, decahydro-4a-methyl-1-methylene-7-(1-methylethenyl)-, [4ar-(4a.alpha.,7.alpha.,8a.beta.)]-	Trp	6.11	14.099
30	1h-cycloprop[e]azulene, decahydro-1,1,7-trimethyl-4-methylene-, [1ar-(1a.alpha.,4a.beta.,7.alpha.,7a.beta.,7b.alpha.)]-	Trp	0.31	14.15
31	Humulene oxide	Trp	5.17	14.249
32	Spiro[4.5]dec-6-en-8-one, 1,7-dimethyl-4-(1-methylethyl)-	Trp	0.48	14.307
33	2-naphthalenemethanol, 1,2,3,4,4a,5,6,7-octahydro-.alpha.,.alpha.,4a,8-tetramethyl-, (2r-cis)-	Trp	0.29	14.373
34	Caryophyllene oxide	Trp	2.62	14.504
35	4,8,8-trimethyl-2-methylene-4-vinylbicyclo[5.2.0]nonane	Trp	1.92	14.591
36	Kauran-18-al, 17-(acetyloxy)-, (4.beta.)-	Trp	1.21	14.669
37	2-naphthalenemethanol, decahydro-.alpha.,.alpha.,4a-trimethyl-8-methylene-, [2r-(2.alpha.,4a.alpha.,8a.beta.)]-	Trp	1.86	14.781
38	1-pentyl-4-(4-propylcyclohexyl)-1-cyclohexene	HC	0.3	14.857
39	3-methyl-5-(2,6,6-trimethyl-1-cyclohexen-1-yl)-1-pentyn-3-ol	Trp	1.45	14.937
40	Longipinane, (e)-	Trp	3.04	15.115
41	2,6,10-cycloundecatrien-1-one, 2,6,9,9-tetramethyl-, (E,E,E)- Zerumbone	Trp	34.54	15.946
42	1-heptatriacotanol	HC	0.58	16.066
43	(-)-5-oxatricyclo[8.2.0.0(4,6)]dodecane,,12-trimethyl-9-methylene-, [1r-(1r*,4r*,6r*,10s*)]-	Trp	1.19	16.297
44	1,11-tridecadiene	HC	0.36	16.55
45	2-methyl-4-(2,6,6-trimethylcyclohex-1-enyl)but-2-en-1-ol	Trp	0.29	16.649
46	2,6,10,15,19,23-hexamethyl-2,10,14,18,22-tetracosapentaene-6,7-diol	Trp	0.41	16.817
47	1h-benzocyclohepten-7-ol, 2,3,4,4a,5,6,7,8-octahydro-1,1,4a,7-tetramethyl-, cis-	Trp	0.14	17.05

48	2,6,10-cycloundecatrien-1-one, 2,6,9,9-tetramethyl-, (E,E,E)-, Zerumbone	Trp	1.5	17.141
49	Hexadecanoic acid, methyl ester	FA	0.89	17.922
50	1H-benzocyclohepten-7-ol, 2,3,4,4a,5,6,7,8-octahydro-1,1,4a,7-tetramethyl-, cis-	Trp	0.22	18.867
51	4,8,13-cyclotetradecatriene-1,3-diol, 1,5,9-trimethyl-12-(1-methylethyl)-	Trp	0.22	18.999
52	9,12-octadecadienoic acid (z,z)-, methyl ester	FA	0.57	20.315
53	9-octadecenoic acid (z)-, methyl ester	FA	0.41	20.408
54	Octadecanoic acid, methyl ester	FA	0.14	20.787
55	Bis(2-ethylhexyl) phthalate	CA	1.16	26.954
	Total		99.99	

*HC: Hydrocarbon, FA: Fatty Acids, Trp: Terpenoids, CA: Carboxylic Acids Derivates

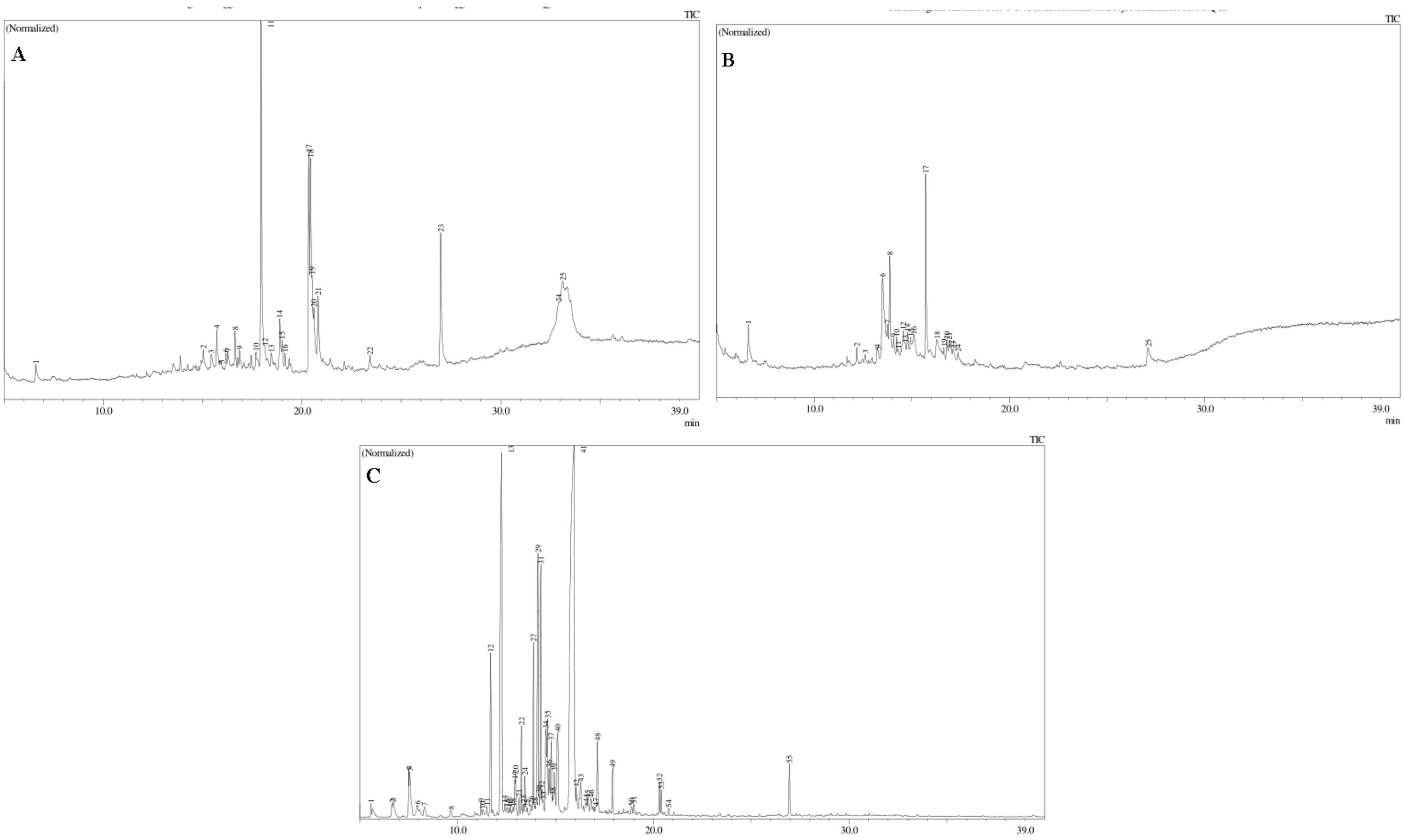

Figure S1. The GC-MS spectral data of *Z. officinale* leaves (A). pseudostems (B) and rhizomes (C) of methanol extract.

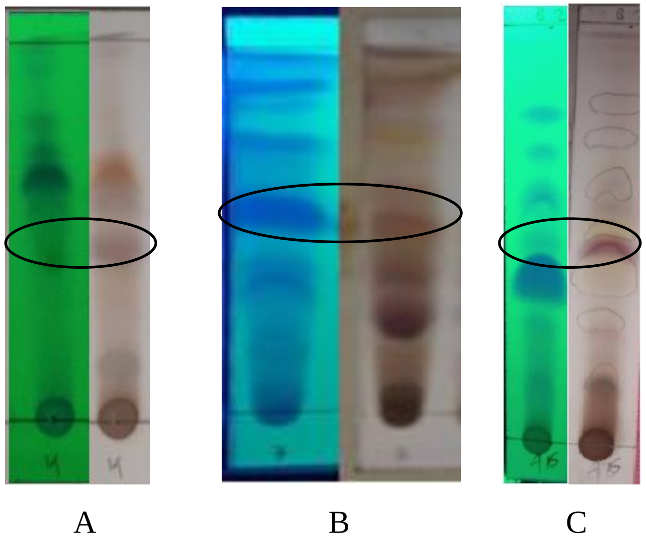

Figure S2. The TLC chromatogram of fraction number 14, 7, and 46 of *Z. officinale* leaves (A). pseudostems (B) and rhizomes (C) of *n*-hexane extract. The spots were detected under UV light 254 nm (left), and after spraying with Liebermann-Burchard reagent (right). The isolated spots were circled out upon the chromatograms.

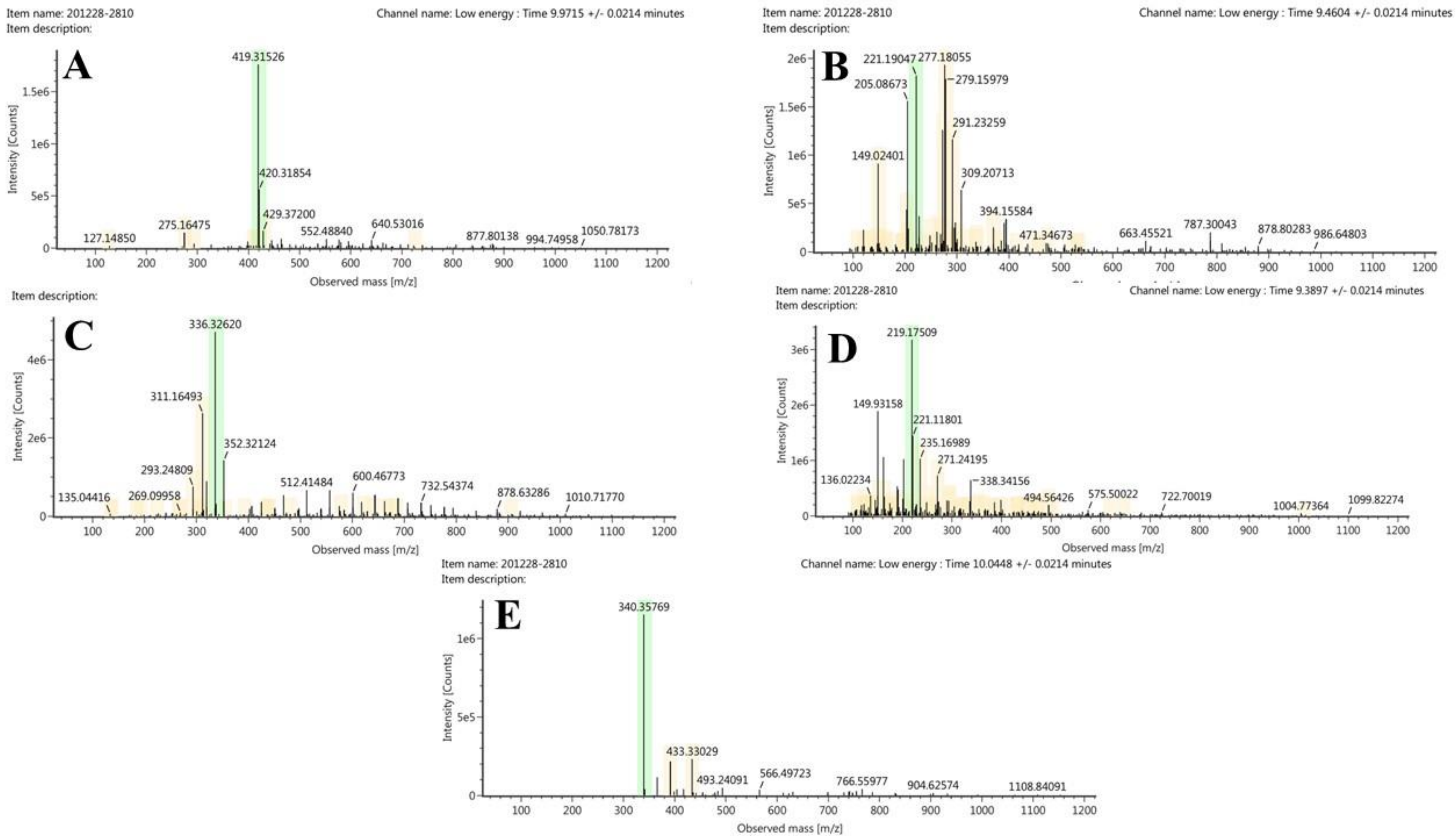

Figure S3. LC-MS/MS spectral data of compounds isolated from leaves of *Z. offiicinale* n-hexane extract. A. (*E*)-Hexadecyl-ferulate, B. Isocyperol, C. N-Isobutyl-(2*E*,4*E*)-octadecadienamide, D. Nootkatone, E. Candidate mass $C_{22}H_{45}NO$

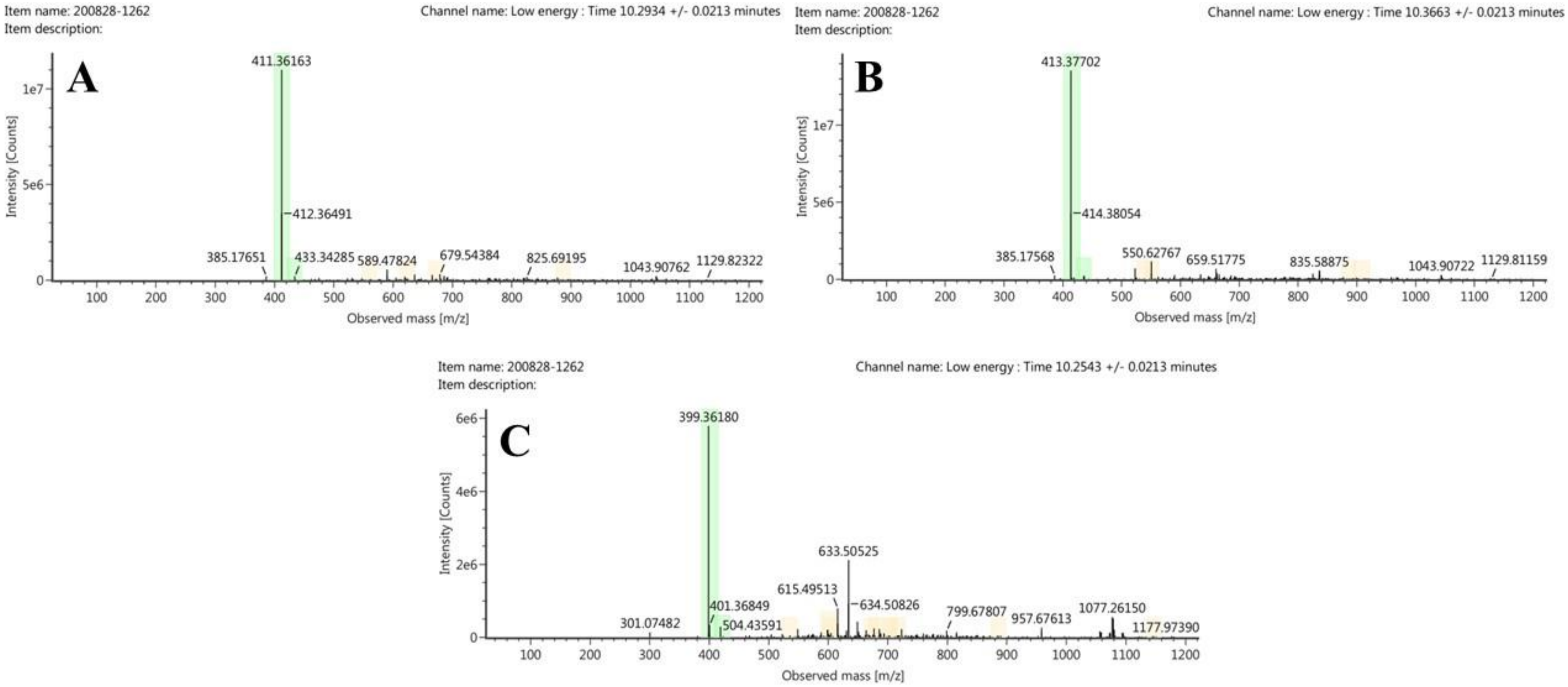

Figure S4. LC-MS/MS spectral data of compounds isolated from pseudostem of *Z. offiicinale* n-hexane extract. A. Spinasterone, B. Spinasterol, C. 24-methylcholesta-7-en- 3*β*-on

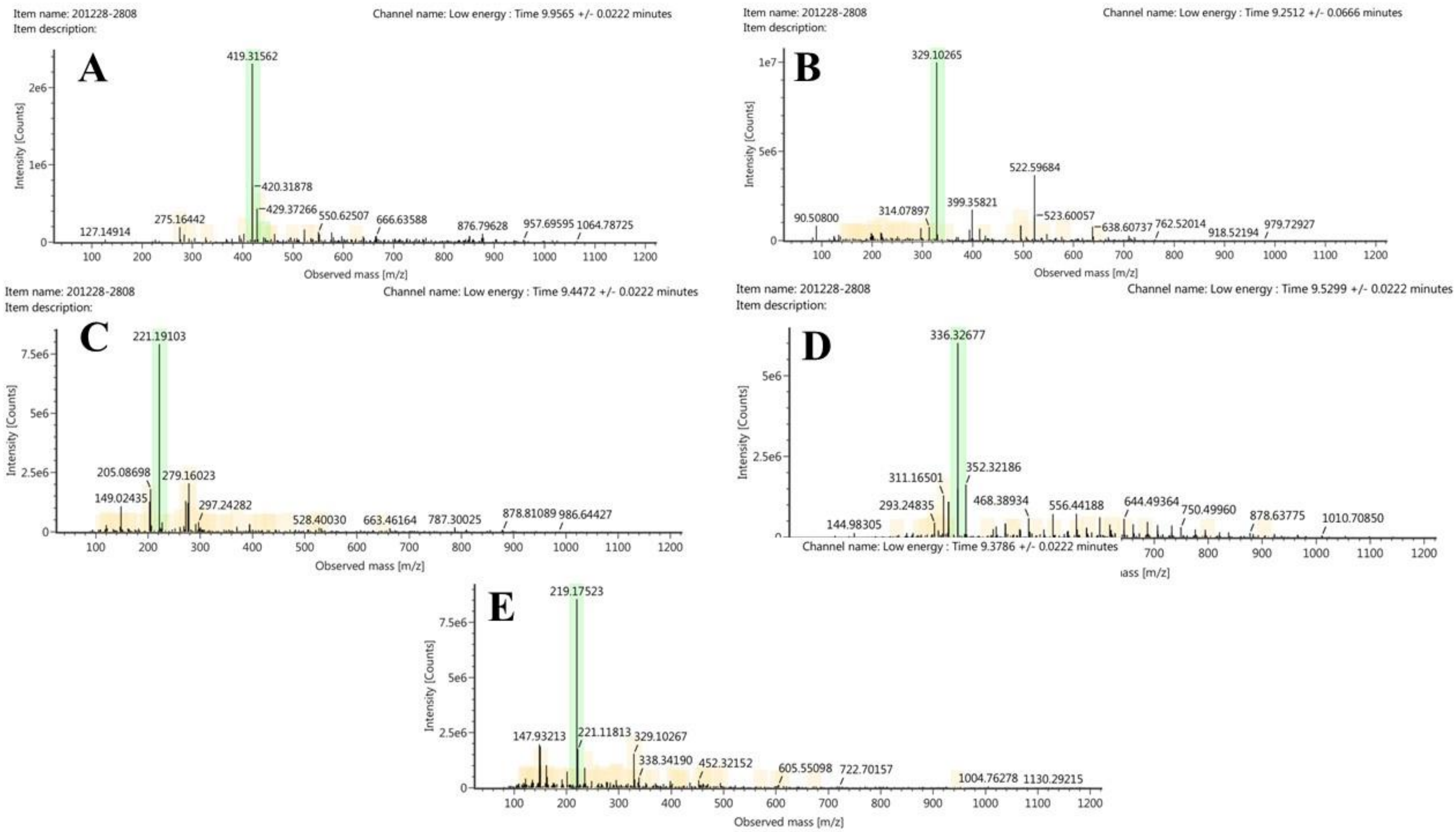

Figure S5. LC-MS/MS spectral data of compounds isolated from rhizome of *Z. offiicinale* n-hexane extract. A. (*E*)-Hexadecyl-ferulate, B. 5-hydro-7,8,2'-trimethoxyflavanone C. Isocyperol, D. N-Isobutyl-(2*E*,4*E*)-octadecadienamide, E. Nootkatone.

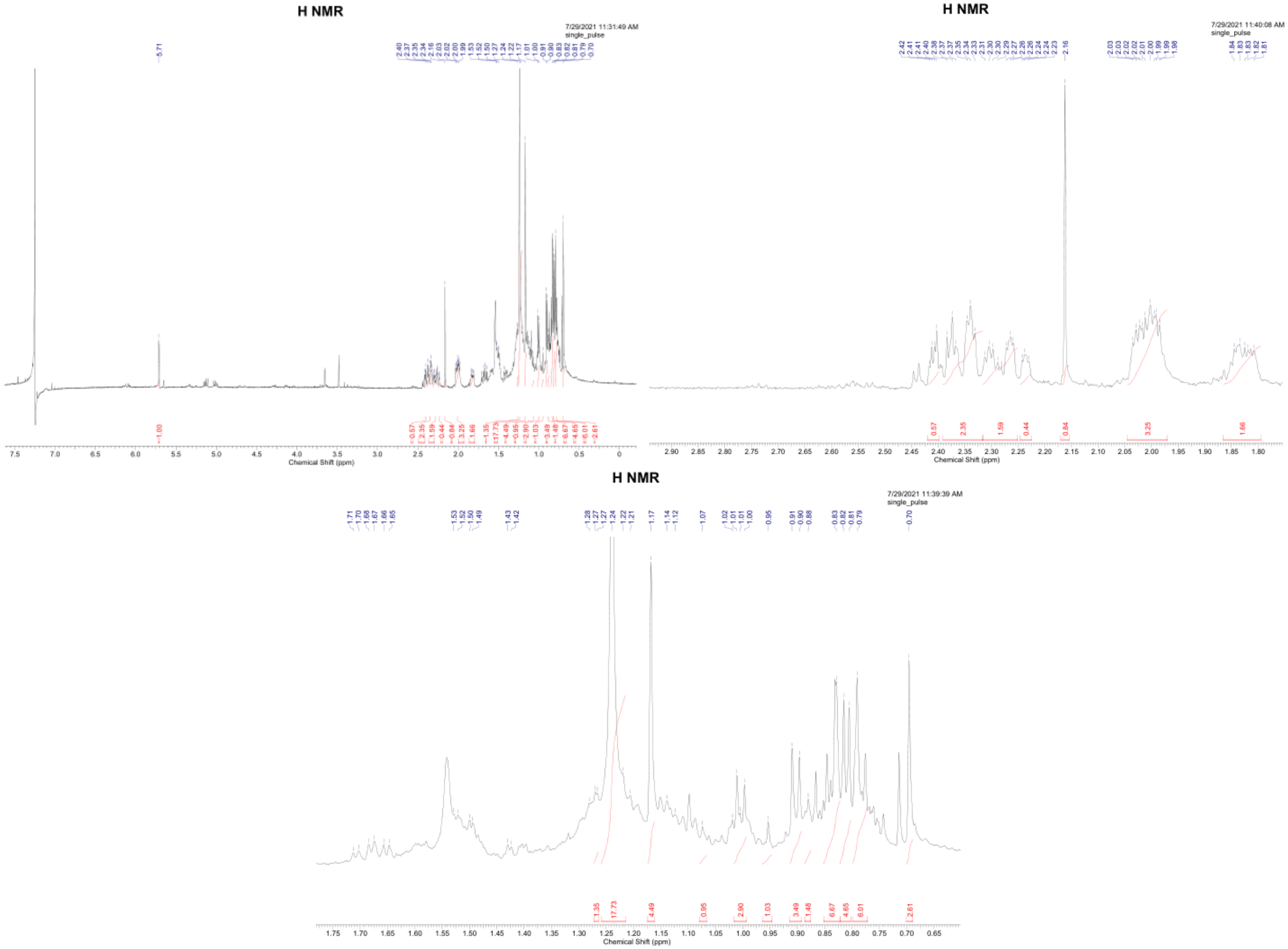

Figure S6. ^{1}H-NMR spectral data of 24-methylcholesta-7-en-3β-on

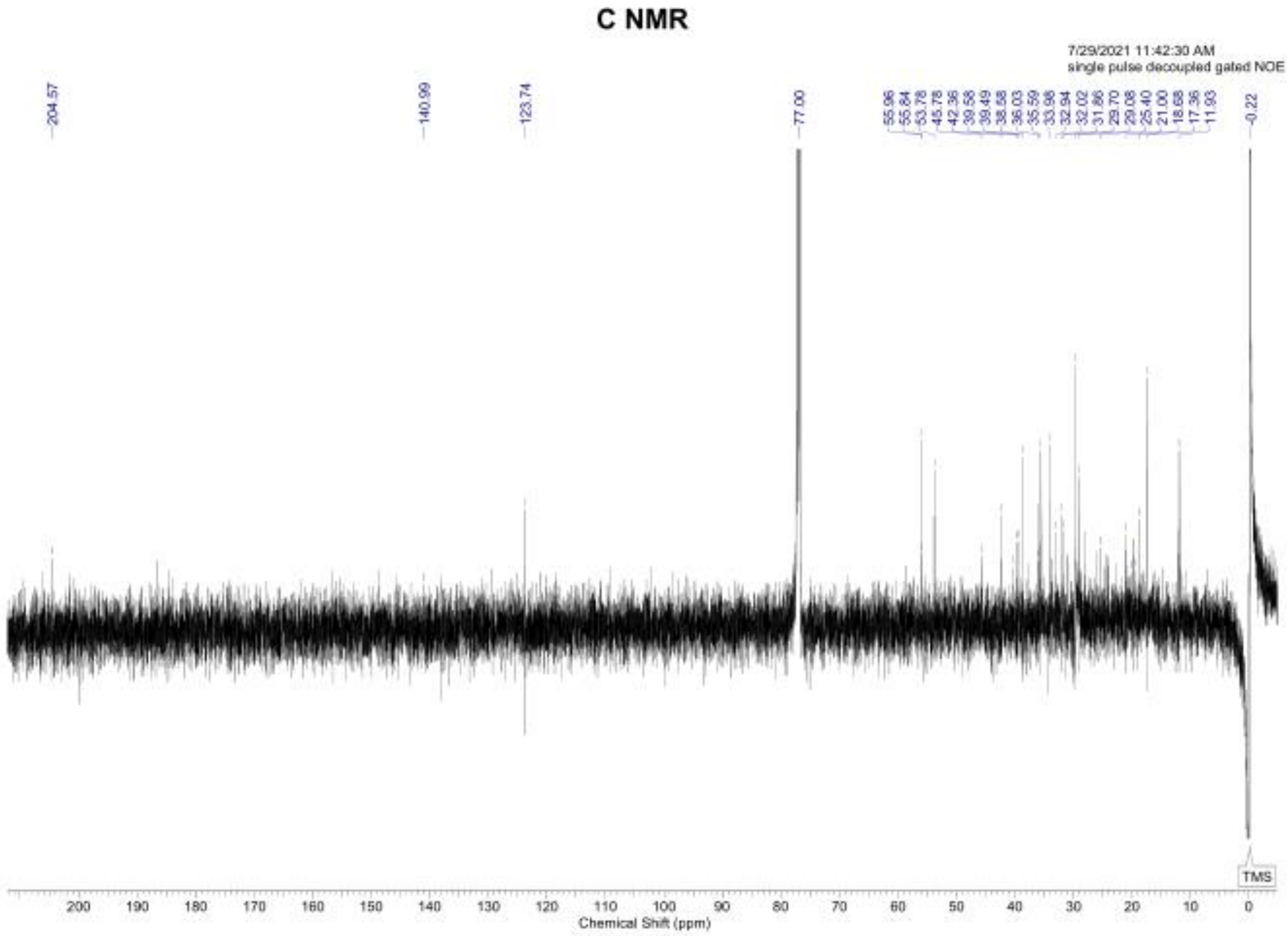

Figure S7. ^{13}C-NMR data of 24-methylcholesta-7-en-3*β*-on

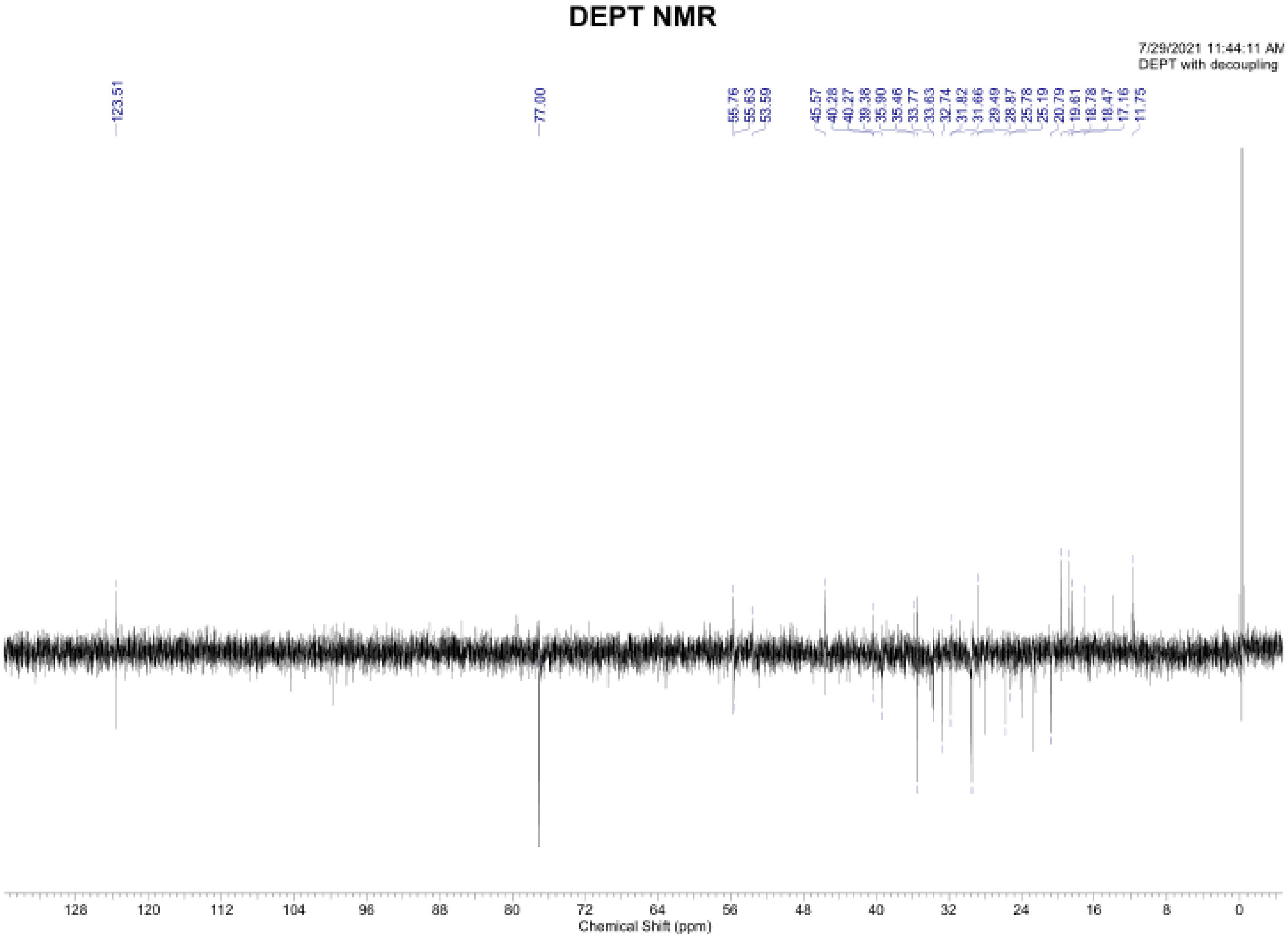

Figure S8. DEPT spectral data of 24-methylcholesta-7-en-3β-on

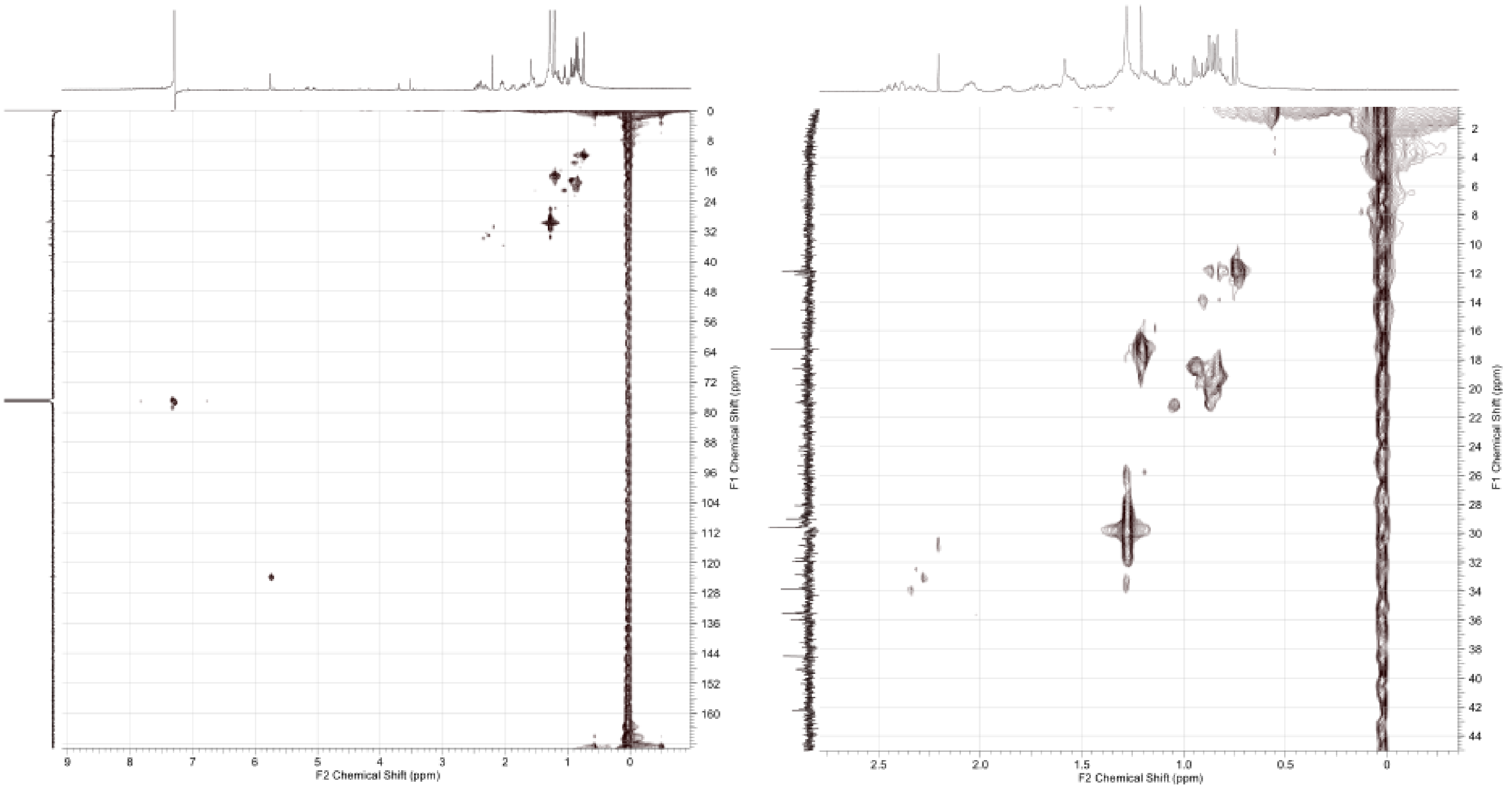

Figure S9. HSQC spectral data of 24-methylcholesta-7-en-3*β*-on

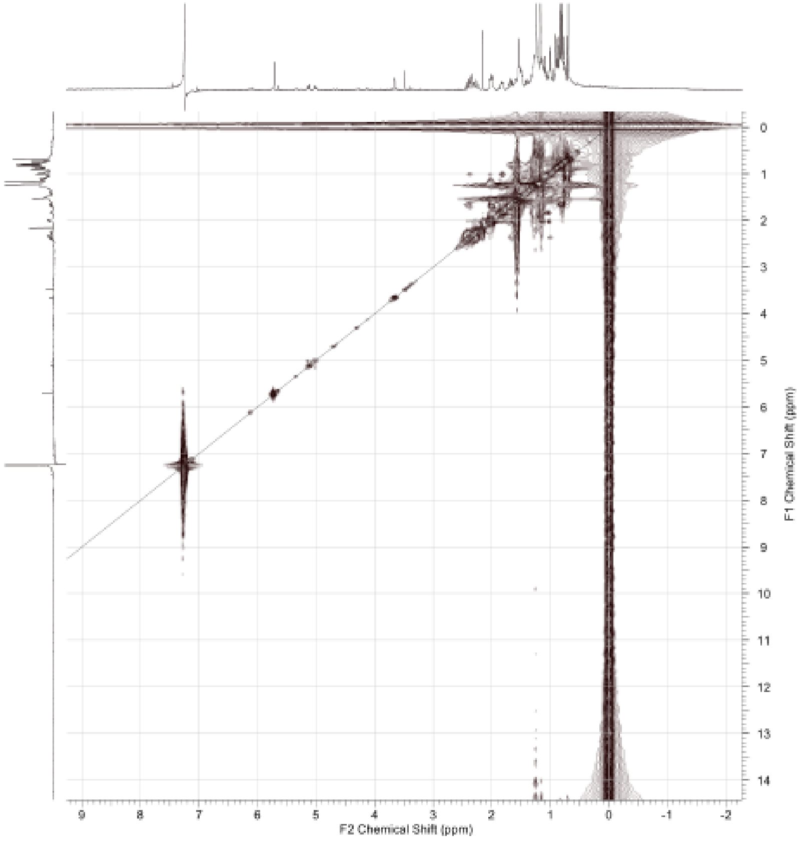

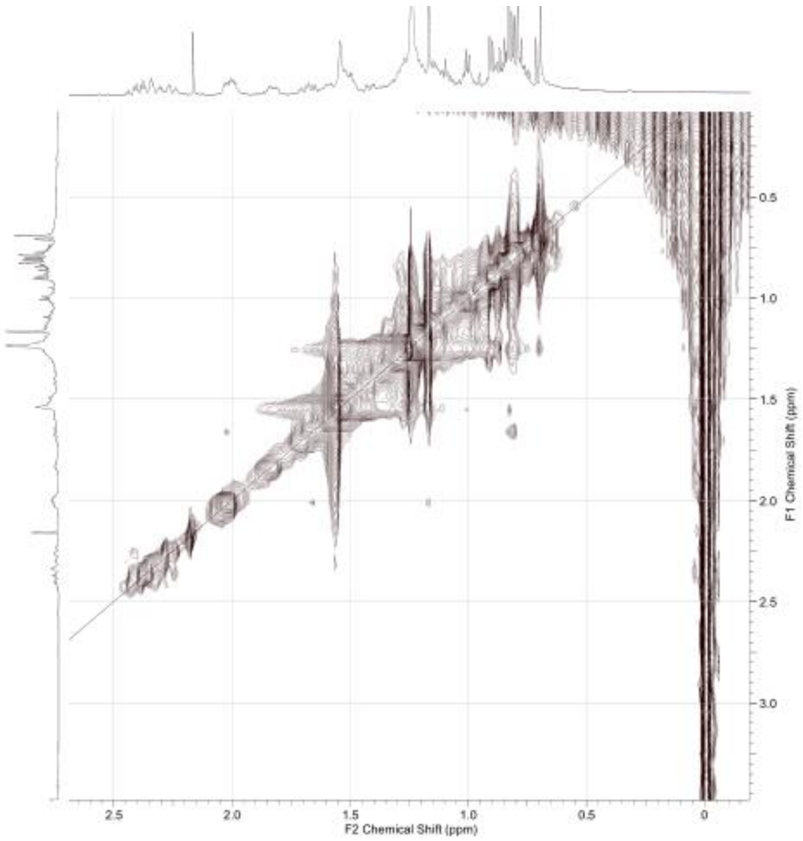

Figure S10. H-H COSY spectral data of 24-methylcholesta-7-en-3β-on

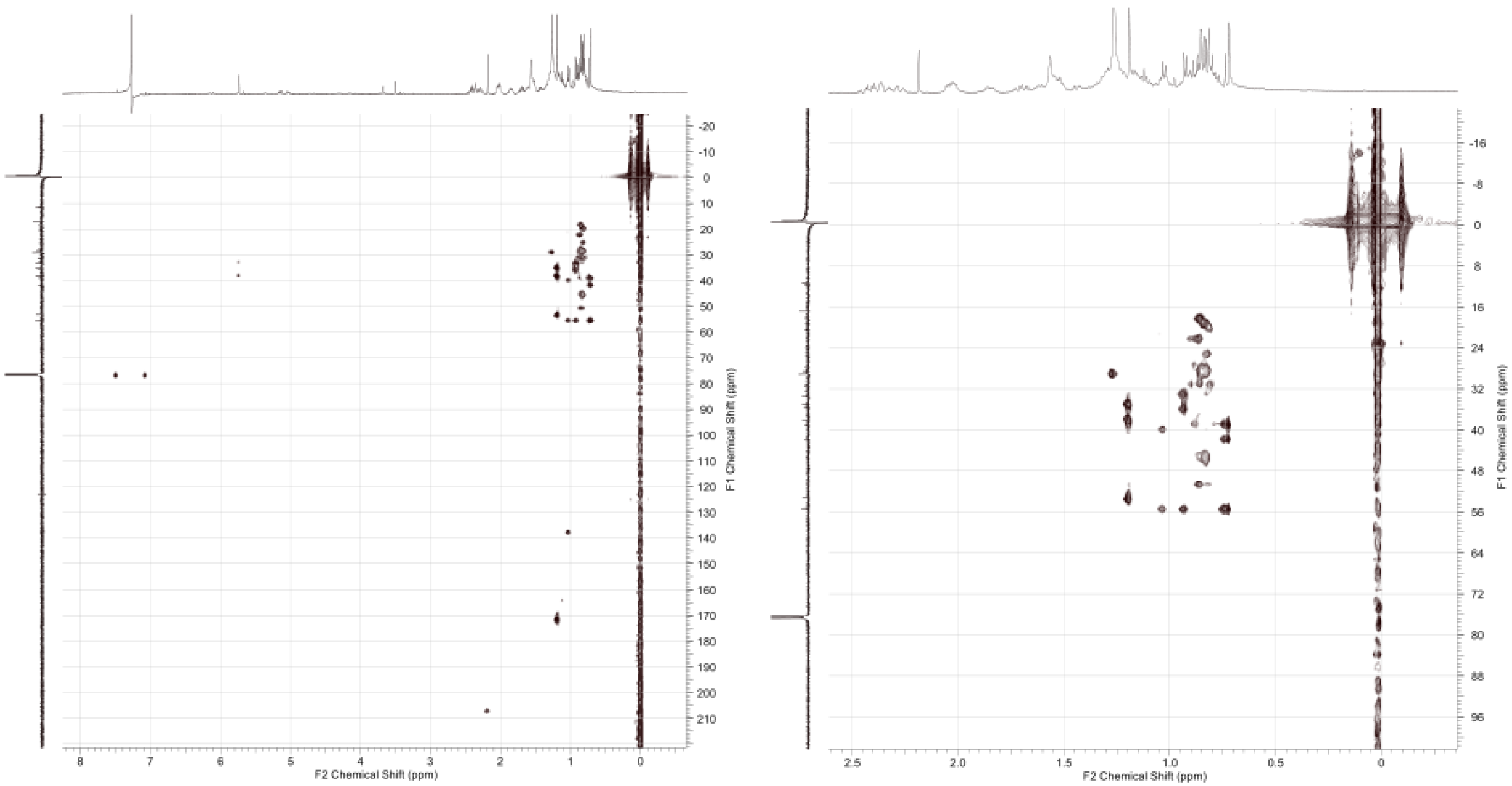

Figure S11. HMBC spectral data of 24-methylcholesta-7-en-3*β*-on
